# Continuous-variable quantum computing on encrypted data

**DOI:** 10.1038/ncomms13795

**Published:** 2016-12-14

**Authors:** Kevin Marshall, Christian S. Jacobsen, Clemens Schäfermeier, Tobias Gehring, Christian Weedbrook, Ulrik L. Andersen

**Affiliations:** 1 Department of Physics, University of Toronto, 60 St. George Street, Toronto M5S 1A7, Canada; 2 Department of Physics, Technical University of Denmark, Fysikvej, 2800 Kongens Lyngby, Denmark; 3 CipherQ, 10 Dundas Street E, Toronto, Canada M5B 2G9

## Abstract

The ability to perform computations on encrypted data is a powerful tool for protecting a client's privacy, especially in today's era of cloud and distributed computing. In terms of privacy, the best solutions that classical techniques can achieve are unfortunately not unconditionally secure in the sense that they are dependent on a hacker's computational power. Here we theoretically investigate, and experimentally demonstrate with Gaussian displacement and squeezing operations, a quantum solution that achieves the security of a user's privacy using the practical technology of continuous variables. We demonstrate losses of up to 10 km both ways between the client and the server and show that security can still be achieved. Our approach offers a number of practical benefits (from a quantum perspective) that could one day allow the potential widespread adoption of this quantum technology in future cloud-based computing networks.

## Abstract (web summary)

Performing computation on encrypted data is a power tool for protecting a client's privacy, but the best solutions achieved by classical approaches are only computationally secure. Here authors present and experimentally demonstrate a quantum protocol to achieve this using continuous variables.

Incredibly, 2.5 quintillion bytes of data are produced in the world every single day. In fact, it has been estimated that >90% of the world's data was created in the past 2 years^1^. Most of these data are stored around the world in data centres and accessed remotely via the cloud. Because cloud computing is operated by third parties (for example, Amazon, Facebook), one of the outcomes of this acceleration in information is the need to better protect our privacy. In principle, the cloud contains various types of data for which security and privacy are essential. For example, an individual's personal data (such as medical records and credit card information), the trade secrets and intellectual property of multinational corporations and sensitive government information (for example, the Central Intelligence Agency bought cloud space from Amazon). Therefore securing a client's privacy in the cloud is one of the core security challenges we face today.

One of the current solutions to this challenge is homomorphic encryption^2^. The requirement for such a solution was first identified in the 1970s by Rivest *et al*.^3^ It was not until over 30 years later that Craig Gentry, in his Stanford PhD dissertation, discovered fully homomorphic encryption^4^. Although there has been much progress in recent years, the best known implementations of fully homomorphic encryption are impractical for today's computers^2^,^5^,^6^,^7^.

One might hope that a generalization of homomorphic encryption to quantum computations would be less demanding. If one restricts the class of quantum operations to be implemented, then it was shown one can hide up to a constant fraction, which can be made arbitrarily close to unity, of the encrypted information while only requiring polynomial overhead^8^. Unfortunately, it has been shown that perfectly secure, deterministic fully homomorphic quantum computation is only possible at the expense of an exponential overhead^9^. One can relax the requirements of quantum homomorphic encryption by allowing further rounds of interaction between the client and server. Such a scheme was first studied by Childs^10^ where he outlined a protocol to allow an individual of limited quantum ability (a client) to delegate a quantum computation to another person (a server) who is in possession of a fully fledged quantum computer. The idea here being that such a computer is only initially available to a select few. Progress towards this direction was demonstrated recently by IBM who made available a small (5 qubit) quantum computer for access in the cloud^11^. The crux of the issue in developing such a protocol lies in the fact that the client wants to hide some subset of her input, the quantum program, and the final result. That such a scheme is even possible is astounding from a classical viewpoint. Seminal work by Broadbent *et al*.^12^ built upon this idea, in the cluster-state framework^13^, to develop the notion of universal blind quantum computing: a protocol that fulfils all three of the above criteria and requires only that the client is able to prepare and send single qubits from a finite set.

The field of delegated quantum computing has since exploded with vast interest in both theoretic advances and experimental demonstrations^14^,^15^,^16^,^17^,^18^,^19^,^20^,^21^,^22^. Unfortunately, accomplishing all three of the client's criteria places stringent requirements on the experimental realization of such protocols. One might hope that by giving up one of these demands, less resources would be required in a physical implementation, while still providing a much needed solution. Interesting work by Fisher *et al*.^23^ showed that if the client and server agree on the program beforehand, then one requires less quantum and classical communication. Such a protocol can be termed quantum computing on encrypted data. This scheme offers the advantage of allowing one to carry out quantum computations on encrypted data with relatively low overhead, namely, generation of random states and classical key updates.

In this paper, we offer an approach to quantum computing on encrypted data that does not require challenging single photon sources or single photon detectors and is based on a different type of substrate known as continuous variables (CVs)^24^,^25^. CVs offer a number of practical advantages over its qubit counterpart: deterministic gate implementation, low cost and affordability of components (such as laser sources and detectors), high detection efficiencies (at room temperature), high rate of information transfer, and the ability to be fully integrated within current telecommunication infrastructures. Here we answer the questions of which gates the client is required to perform, how much classical communication is required between the client and server and how many classical/quantum operations are needed per gate in our protocol. Furthermore, we provide proof-of-principle experimental results that highlight the effect of loss equivalent to over 10 km of fibre transmission at a telecom wavelength.

## Results

### Theory

**Figure 1:**
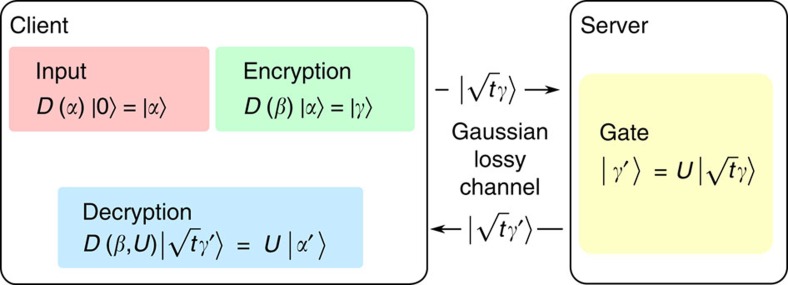
Protocol for quantum computing on encrypted data. The three stages of our protocol: encryption, gates, and decryption, are illustrated for a coherent state input where we include a transmission step in both directions between the client and server. Input: a displaced vacuum state is prepared as indicated by the red box. Encryption: Next, in the green box, a random displacement is applied to the initial state as an encryption procedure. Channel: The state is transmitted, denoted by a right arrow, over a Gaussian lossy channel to the server (transmission *t*). Gate: The server applies the desired unitary (a Gaussian displacement or squeezing operation in our experiment), indicated by the yellow box. Channel: The state is sent back over the Gaussian lossy channel to the client, shown in the figure as a left arrow. Decryption: The client applies a decryption operation to retrieve the final output state as described in the blue box.

Our protocol for computing on encrypted CVs consists of three stages (cf. Fig. 1): an encryption stage, a program stage, and a decryption stage. We will now elaborate in more detail. First, the client performs an encryption operation on their desired input to limit the amount of information the server can obtain about the initial state. The state is then sent to the server, who performs a predetermined set of gates known to both parties (corresponding to the program needed to be performed). Finally, the state is sent back to the client who is able to perform a decryption operation that recovers the output of the desired computation. To discuss the encryption operation, we first define the Heisenberg–Weyl operators^25^ 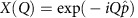 and 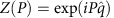, as well as the displacement operator 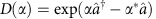 where 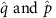 are the canonical amplitude and phase operators, respectively, which obey Heisenberg's uncertainty relation 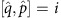. The annihilation and creation operators are denoted by 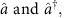 respectively, and are defined by 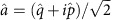 and its adjoint. Consider the action of applying a random displacement in phase space to the input state; intuitively speaking, if the displacement is chosen randomly then the state will look mixed and smeared out over all of phase space to somebody unaware of the displacement parameters. This can be made rigorous by invoking the fact that 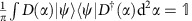 holds for any normalized state 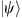. This shows us that averaging the displacements applied to any normalized state, over the entire complex plane, results in a quantity proportional to the identity. In reality, a uniform distribution of displacements over 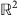 is unphysical and we must replace this with a function that dies off sufficiently fast in order to adhere to some energy threshold. For a fixed energy, a Gaussian distribution will maximize the entropy of the resulting state^26^ and thus we restrict ourselves to Gaussian distributions. This will potentially give the server the capability of extracting some information about the input state, but the amount will be bounded based on the width of the Gaussian. A formal security analysis of our protocol, in the limit of finite squeezing and displacements, remains an open problem.

**Figure 2:**
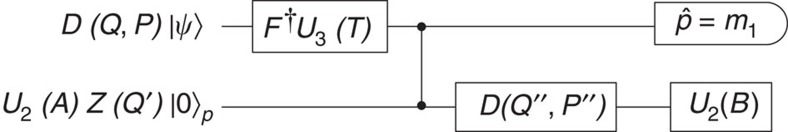
Server implementing a cubic gate. A quantum circuit demonstrating the implementation of *U*_3_(*T*) on a desired qumode held by the server (top wire). The client is able to correct for the *U*_2_(−3*QT*) gate in the decryption operation for *U*_3_(*T*) on-the-fly by sending an ancilla qumode (bottom wire). By choosing *A* and *Q*′ randomly and keeping these parameters hidden, the client is able to have the server implement the decryption without divulging information about the encryption parameter *Q*. The parameters *Q″* and *P″* are defined in Supplementary Note 1.

We now turn our attention to the server who is asked to perform some known algorithm on the encrypted data, where, in principle, the algorithm corresponds to a universal set of quantum gates. To show that the server can perform such a computation, it suffices to show that we can implement a universal set of gates. Namely, we need to show that the set 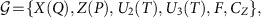 where 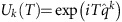, 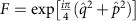 and 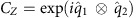 can be implemented and decrypted with an appropriately chosen operation. Note that of the gates in this set only the *U*_3_(*T*) gate^27^,^28^ is not Gaussian, and we only require this one non-Gaussian gate in order to achieve universal quantum computation^29^. However, it is prudent to note that non-Gaussian operations are very challenging to implement and that there remain many practical challenges in devising a robust quantum computer based on CVs^29^. Except for *U*_3_, all of these operations have decryption operators that correspond to displacements (cf. Table 1), and this allows for the straightforward composition of gates. To make it clearer, we consider a simple example, namely, the *Z*(*T*) gate. The encryption operation *D*(*Q*, *P*) consists of a translation in both the amplitude and phase quadratures and it can be decomposed as a sequence of an *X*(*Q*) as well as a *Z*(*P*) gate, the latter commutes with *Z*(*T*) and so will simply slide through the gate. Consider the application of the *X*(*Q*) gate: this gate slides through the *Z*(*T*) gate up to a phase as *X*(*Q*)*Z*(*T*)=*e*^−*iQT*^*Z*(*T*)*X*(*Q*). Thus we can construct a decryption operation as


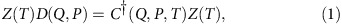


where *C*(*Q*, *P*, *T*)=exp[*i*(*QP*/2−*QT*)]*X*(−*Q*)*Z*(−*P*) is the decryption gate which, when applied, will undo the effect of the initial encryption operation. However, the *U*_2_ decryption gate present in the *U*_3_ operation does not easily slide through the Fourier gate *F*, and thus we must have the server correct for this on-the-fly; this is possible in a manner similar to the discrete-variable protocol presented in ref. 23. To perform *U*_3_(*T*), the client instead sends the server two modes, the first of which is the encrypted state and the second being the state *U*_2_(*A*)*Z*(*Q*′)|0〉_*p*_, where *A* and *Q*′ are chosen randomly and |0〉_*p*_ denotes a momentum eigenstate; finite squeezing does not present any issues other than the ones normally associated with teleportation, namely, the introduction of extra Gaussian noise^30^. The server is then able to implement *U*_3_(*T*) as shown in the circuit in Fig. 2. After the client sends both modes and the value of *B*, the server performs the desired *U*_3_(*T*) gate after application of the inverse Fourier gate, before interacting the two modes with a controlled phase gate, indicated by the vertical line. The server then measures the first mode and after performing the *U*_2_(*B*) gate obtains the desired state *U*_3_(*T*)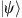 on the remaining mode, up to displacement corrections, provided that *B*=−3*QT*−*A*. One can view this decomposition of −3*QT* as an additive white Gaussian noise channel where the input is modulated by a noise source *A*; as the variance of *A* increases the channel capacity approaches zero and thus the server obtains no information about the encryption parameter *Q*. Note that the additional corrections depend on the value *m*_1_*A* and so the server must communicate the value of *m*_1_ obtained to the client, thus requiring one round of classical communication for the full implementation. In the limit of ideal teleportation and infinite variance in the Gaussian probability density functions governing the selection of *A* and *Q*′, it can be shown that this interactive protocol does not compromise the encryption; the proof follows identically to that in ref. 23. A proof in the presence of both finite squeezing and displacements remains an open challenge.

We discuss the implementation of this protocol in more detail as well as a discussion of the decryption operations for each gate in the universal set in Supplementary Note 1. We provide additional details in Supplementary Information, in particular: how to compose gates (Supplementary Note 2), the effects of transmission (Supplementary Note 3) and imperfect encryption (Supplementary Note 4), an entanglement-based analogue (Supplementary Note 5), the use of channel estimation (Supplementary Note 6), and limitations on squeezing (Supplementary Note 7).

### Experiment

The CV quantum gates associated with linear phase space displacements and squeezing transformations allow for an experimental test of quantum computing on encrypted data solely based on Gaussian quantum states and Gaussian operations. Such operations can be performed with high fidelity within the field of CV quantum optics. In the following, we thus use Gaussian displacement and squeezing operations to test the basic principles of quantum computing on encrypted data, that is, we implement a server performing first *Z* and *X* gates and second a squeezing gate, related to the *U*_2_ gate.

**Figure 3:**
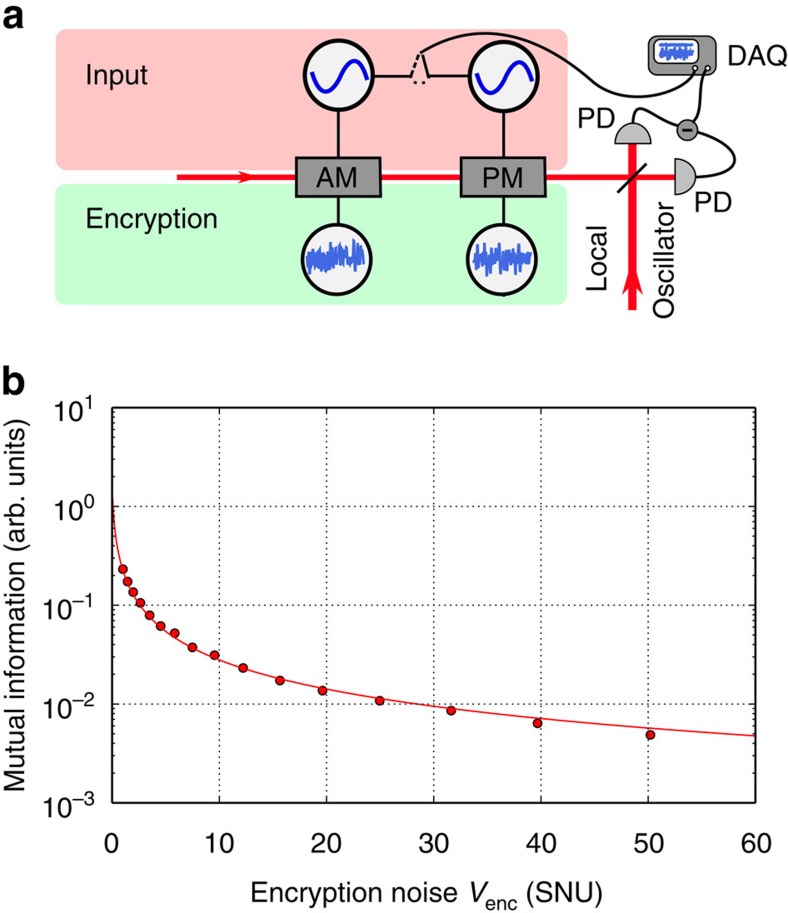
Effectiveness of encryption. (**a**) Experimental setup using electro-optical amplitude and phase modulators (AM and PM, respectively) driven by Gaussian white noise sources to generate ensembles of encrypted coherent states. Both the input noise and the homodyne output were demodulated at 10.5 MHz and recorded by a data acquisition system (DAQ). PD: photo detectors. (**b**) The mutual information *I*(server_enc_ : client_in_) for a coherent state chosen according to a Gaussian alphabet with variance *V*_in_=0.6 SNU, which is then encrypted with a varied encryption variance *V*_enc_. For a fixed distribution of input states, the plot shows how an increased encryption noise decreases the server's knowledge of the inputs. Error bars are smaller than the point size.

We start by testing the effectiveness of the encryption operation by using the experimental setup shown in Fig. 3a. Quantum information at the location of the client was generated in the form of a coherent state of light |*ϕ*〉=|*α*〉. To test the protocol for many different coherent state excitations simultaneously, we produced an ensemble of coherent states by means of a set of electro-optical modulators (EOMs), thereby preparing the Gaussian ensemble 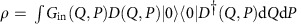 where *G*_in_(*Q*, *P*) is a Gaussian probability density function with variance *V*_in_. This information was then encrypted by applying a randomized phase space displacement onto the coherent state ensemble using the same two EOMs driven by two independent Gaussian white noise sources with equal variances *V*_*Q*_=*V*_*P*_=*V*_enc_ for the amplitude (*Q*) and phase (*P*) quadratures. This encryption noise results in an encrypted state 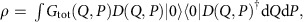 where *G*_tot_(*Q*, *P*) is a Gaussian distribution with a total variance of *V*_in_+*V*_enc_. We measured the encrypted quantum states with homodyne detection and recorded the correlations between the measurements and the input signal. Using these correlations, we calculate the mutual information as plotted in Fig. 3b. The solid line is a theoretical prediction given by


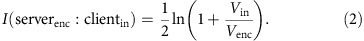


For efficient encryption, the quadrature correlations and thus the mutual information between the encrypted state received by the server and the input state prepared by the client should be vanishingly small. From the plot, we clearly see the effects of a finite encryption variance.

**Figure 4:**
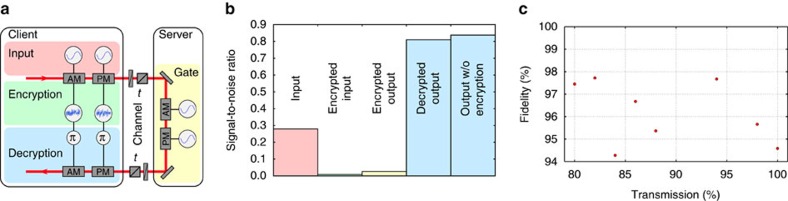
Server implementing *Z* and *X* gates. (**a**) Experimental setup. Ensembles of encrypted input states were generated by two EOMs driven by two Gaussian white noise sources. The encrypted quantum states were then sent to the server through a channel with transmittivity *t* simulated by a half-wave plate and a polarizing beam splitter. The server performed ensembles of displacements by using a second pair of modulators driven by independent Gaussian white noise generators. After sending the quantum state back to the client, the output of the quantum computer was decrypted by applying phase shifted modulations using the encryption noise only known to the client. (**b**) The signal-to-noise ratios of the phase quadrature measured by homodyne detection behind each stage of the protocol. An ensemble of coherent input states and gate displacements were used and the transmissivity of the channels was set to 1. The output of the homodyne detector was recorded by a spectrum analyser measuring zero span around 10.5 MHz with a resolution bandwidth of 300 kHz and a video bandwidth of 30 Hz. A small input state was prepared (red) and subsequently encrypted (green). Then a small displacement was performed by the server (yellow), acting as the gate. Afterwards the state was returned to the client for decryption (blue), which yields the output of the quantum computation. For comparison, we have recorded the outcome without encrypting the input (upper trace). The difference between these is indicative of the loss for signal-to-noise ratio from imperfect decryption. (**c**) Fidelities between the output state ensembles using quantum computation on encrypted states and using quantum computation on plain-text states versus the channel transmission *t*. Statistical error bars are smaller than the point size. The variation in the fidelities comes mainly from systematic errors in the fine tuning of the phase and gain settings of the decryption noise for optimal decryption.

We first implement the *Z* and *X* displacement gates as illustrated in Fig. 4a. The protocol was performed with a Gaussian alphabet of coherent states with variance *V*_in_=0.28 shot noise units (SNU) embedded in encryption noise of *V*_enc_=31 SNU. For this particular encryption, the mutual information is *I*=0.005 bits per use. The *Z* and *X* gates were tested for a symmetric Gaussian distribution of displacements with variance *V*_gate_=0.6 SNU. This results in the state *ρ* after the computation having a total variance of *V*_gate_+*V*_in_+*V*_enc_.

Finally, the state is sent back through a lossy channel to the client who is decrypting the state using two EOMs driven by noise that was optimally anti-correlated with the noise used for encryption. The final state is then ideally given by *D*(*Q*, *P*)|*ϕ*〉, but owing to imperfections there is some residual noise from the encryption protocol and thus we must consider the output state *ρ* with a variance of *V*_in_+*V*_gate_+*V*_res_. For the presented measurement, the residual noise was *V*_res_=0.072 SNU. To visualize the evolution of the information content at different stages of the scheme, in Fig. 4b, we plot the signal-to-noise ratios (SNRs) of a single quadrature after each stage of the protocol. It is clear from these numbers that the amount of information in the encrypted state is close to zero and that the decryption operation is almost ideal. For further quantification, we show in Fig. 4c the fidelity between the ensembles of output states of the quantum computation using encrypted states and plain-text states. The fidelities are >97% for all measured transmission values.

**Figure 5:**
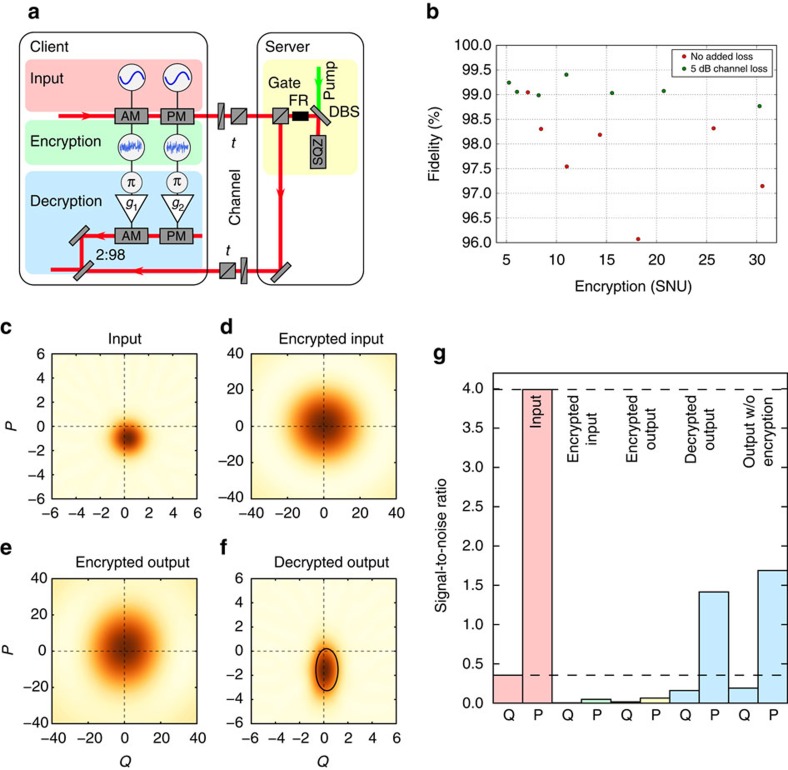
Server implementing a squeezing gate. (**a**) Experimental setup. The squeezing gate was implemented by injecting the input states into a squeezed light source made of a periodically poled potassium titanyl phosphate in a linear cavity. After squeezing, the output state was sent back to the client who decrypted it by interfering the mode with another beam that was modulated with *π*-phase shifted encryption noise at a 2% tap-off beam splitter. Owing to squeezing the amplitude quadrature, the decryption noise was amplified differently in the two quadratures with gains *g*_1_ and *g*_2_ depending on the squeezing strength. More details can be found in Supplementary Methods. (**b**) The fidelity between the output states of the computation on encrypted and plain-text states with both 100% channel transmission and 5 dB transmission loss corresponding to optical loss in 10 km fibre at telecom wavelength. Statistical error bars are smaller than the point size. The variation of the fidelities comes mainly from systematic errors in the fine tuning of the decryption and system drifts. (**c**–**f**) Reconstructed Wigner functions in phase space for each step in the protocol with a coherent input state and the squeezing gate measured by homodyne detection after each step at a side-band frequency of 10.5 MHz. The black circle in **f** denotes the full width at half maximum (FWHM) of the ideal squeezing ellipse. (**g**) Shows the signal-to-noise ratios in the Q and P quadratures for the states shown in panels **c**–**f** as well as for an output state that was not encrypted during computation. The dashed lines indicate the signal-to-noise ratio of an output state using an ideal gate and no channel loss.

We now turn to the implementation of the squeezing gate, which is defined as 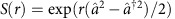, where *r* is the squeezing parameter. It is directly related to the *U*_2_(*T*) gate by two additional phase shifts and a suitable transformation^31^ between *T* and *r*, see Supplementary Note 8 for a full justification. Figure 5a shows the experimental setup. In contrast to the implementation of the displacement gates, we used a single coherent excitation rather than ensembles of coherent input states. The squeezed-light source was based on parametric down-conversion in a periodically poled potassium titanyl phosphate crystal placed in a linear cavity. In our realization of the gate, we directly squeezed the (encrypted) input state in the squeezed-light source. We note that this constitutes the first demonstration of an in-line squeezing transformation of quantum information. Previous demonstrations have relied on off-line squeezed states^31^. A full Wigner function illustration is presented in Fig. 5c–f measured by homodyne detection after each of the four steps. The state after the squeezing gate is shown in Fig. 5e, and it is clear that the squeezing operation is hardly visible as a result of the encryption noise. Finally, the transformed state is decrypted through displacements at the client, thereby revealing the output state of the server which is displayed in Fig. 5f. The squeezing transformations of the first and second moments of the state are now clearly visible.

We quantify the performance of the squeezing protocol on encrypted data by computing the fidelity between the state retrieved by the client after decrypting the computed input state and the state received by the client when no encryption was used. This comparison is carried out under the variation of the amount of encryption noise for both no transmission loss and loss corresponding to 10 km fibre propagation at a telecom wavelength, equivalent to 5 dB transmission loss. This loss is implemented with a half-wave plate and polarizing beam splitter combination. The results are presented in Fig. 5b. The variation of the fidelities, as was also observed for the displacement gates, comes mainly from systematic errors in the fine tuning of the decryption and system drifts. The deviation from unity fidelity is mainly caused by imperfect reconstruction of the Wigner functions and system drifts as well as non-ideal decryption owing to a small amount of decorrelation between the encryption and decryption noise. Despite these imperfections, the fidelity is close to unity and stays >98.5% for all parameters. In general, the fidelities for the loss case are a bit higher owing to the fact that the loss forces the decrypted states closer to the vacuum state. The measured high fidelities show that performing quantum computing on encrypted states rather than on plain-text states is indeed feasible.

To quantify the performance of the implemented remote gate itself, we display in Fig. 5g the SNRs of the *Q* and *P* quadratures for the different steps of the protocol. Ideally, that is, without channel loss, without loss in the gate and with perfect decryption, the squeezing gate will preserve the SNR as indicated by the two dashed lines. For the output state, we display the SNRs for both with and without encryption, showing a small decrease in SNR if encryption is used. The remaining reduction of the SNR in comparison to the ideal gate is mainly due to optical loss, that is, about 2.2 dB for the gate, including the squeezer and the supply optics. A more detailed loss analysis can be found in Supplementary Methods.

## Discussion

We have developed a continuous-variable protocol for quantum computing on encrypted variables where we required a baseline of only two uses of a quantum channel: one use for the input and another for the output. We required one additional round of classical communication in each direction and one additional use of the quantum channel to implement a cubic phase gate *U*_3_(*T*), while Gaussian gates can be implemented with no communication cost. The client needs only be capable of performing displacements to both encrypt and decrypt, except when they perform a *U*_3_(*T*) gate the client must be capable of implementing a *U*_2_(*A*) gate as well. Alternatively, one could run an equivalent entanglement-based version of this protocol, which relies on teleportation^32^. To achieve high-fidelity teleportation, one could use a hybrid teleportation scheme such as that of ref. 33.

In this paper, we also studied how to compose gates for encryption and decryption, the effects of transmission and imperfect encryption, an entanglement-based analogue and the use of channel estimation as well as the limitations on squeezing in the cubic phase gate. We have experimentally demonstrated our scheme in performing both displacements and online squeezing operations on an alphabet of coherent states and studied the resulting performance in terms of the fidelity. Finally, to the best of our knowledge, this is the first time quantum computing on encrypted data has been generalized to CVs as well as the first proof-of-principle demonstration of any form (qubit or qumode) of secure delegated quantum computing over a lossy channel. Extending this protocol to long distances will inevitably require quantum-repeater technologies to preserve the encrypted states^34^. We hope our results will help lay the ground work for future theoretical explorations and experimental demonstrations similar to those for quantum key distribution, such as free space and field demonstrations.

## Methods

### State preparation and gate operations

In all experiments, state preparation was carried out using a coherent continuous-wave 1,064 nm laser beam as a carrier. The investigated quantum states were prepared at 10.5 MHz sidebands relative to the carrier, where the spectrum was shot-noise limited. Coherent states were prepared in these sidebands by injecting the laser beam into a pair of EOMs, which were driven with 10.5 MHz sinusoidal signals. Thermal states were prepared by applying two independent white noise signals around 10.5 MHz to a pair of EOMs. Squeezed states were prepared using a semi-monolithic cavity with a temperature-stabilized periodically poled potassium titanyl phosphate crystal as a non-linear medium, pumped by 532 nm light. The cavity length was locked using the Hänsch–Couillaud locking scheme^35^. The relative phase between the pump and the quantum state carrier beams was locked through a 36.7 MHz sideband lock applied onto the carrier beam by the EOM, which also produces the input state in the protocol. See Fig. 5.

### Measurements

Output states were measured using scanned homodyne detection. The detector's output was mixed with an electronic local oscillator at 10.5 MHz in an analogue mixer. The down-mixed output was amplified by an amplifier with adjustable gain and low-pass filtered at 1 MHz. The filtered signal was digitized with a 14 bit digital-to-analogue converter at a rate of 5 MHz.

### Data processing

The digitized signal was filtered with a 10 kHz high-pass filter to suppress 50 Hz fluctuations in the measurements originating from the voltage supply. The density matrices of the measured states were reconstructed from the digitized and filtered data through the use of maximum likelihood and filtered back-projection algorithms^36^, depending on the mean photon number of the state. The fidelity was estimated from the reconstructed density matrices, using the equation^25^


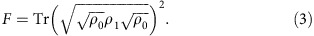


SNRs were determined from the estimated variances and mean values of the reconstructed states.

### Data availability

Data and data processing scripts are available from the corresponding authors upon request.

## Additional information

**How to cite this article:** Marshall, K. *et al*. Continuous-variable quantum computing on encrypted data. *Nat. Commun.* **7**:13795 doi: 10.1038/ncomms13795 (2016).

**Publisher's note**: Springer Nature remains neutral with regard to jurisdictional claims in published maps and institutional affiliations.

## Supplementary Material

Supplementary Information Supplementary Figures 1-9, Supplementary Table 1, Supplementary Notes 1-8, Supplementary Methods and Supplementary References.

## Figures and Tables

**Table 1:** Quantum computing gates and corresponding decryption operations.

**Gate**	**Correction**
*Z*(*T*)	*X*(−*Q*)*Z*(−*P*)
*X*(*T*)	*X*(−*Q*)*Z*(−*P*)
*U*_2_(*T*)	*X*(−*Q*)*Z*(−2*QT*−*P*)
*U*_3_(*T*)	*X*(−*Q*)*Z*(3*Q*^2^*T*−*P*)*U*_2_(−3*QT*)
*F*	*X*(*P*)*Z*(−*Q*)
*C*_*Z*_	*X*_1_(−*Q*_1_)*Z*_1_(−*Q*_2_−*P*_1_) ⊗ *X*_2_(−*Q*_2_)*Z*_2_(−*Q*_1_−*P*_2_)

The decryption operations corresponding to each gate are listed, up to a phase, for the corresponding encryption operation *D*(*Q*, *P*) for single mode gates and *D*_1_(*Q*_1_, *P*_1_)*D*_2_(*Q*_2_, *P*_2_) for two-mode gates.

## References

[b1] IBM. What will we make of this moment? *2013 IBM Annual Report*, 2–13 (2013).

[b2] Naehrig M., Lauter K. & Vaikuntanathan V. in *Proceedings of the 3rd ACM Workshop on Cloud Computing Security Workshop, CCSW'11* 113–124ACM (2011).

[b3] Rivest R., Adleman L. & Dertouzos M. in *Foundations of Secure Computation* Vol. 4 (eds DeMillo, R., Dobkin, D., Jones, A. & Lipton, R.) 169–180 (Academic Press, New York, 1978).

[b4] Gentry C. in *Proceedings of the Forty-first Annual ACM Symposium on Theory of Computing, STOC ‘09* 169–178ACM (2009).

[b5] Brakerski Z. & Vaikuntanathan V. in *Proceedings of the 2011 IEEE 52Nd Annual Symposium on Foundations of Computer Science, FOCS' 11* 97–106IEEE Computer Society (2011).

[b6] Brakerski Z., Gentry C. & Vaikuntanathan V. in *Proceedings of the 3rd Innovations in Theoretical Computer Science Conference* 309–325ACM (2012).

[b7] Gentry C., Halevi S. & Smart N. P. Homomorphic evaluation of the AES circuit. Preprint available at https://eprint.iacr.org (2015).

[b8] Tan S.-H., Kettlewell J. A., Ouyang Y., Chen L. & Fitzsimons J. F. A quantum approach to homomorphic encryption. Sci. Rep. 6, 33467 (2016). 27658349 10.1038/srep33467 PMC5034262

[b9] Yu L., Pérez-Delgado C. A. & Fitzsimons J. F. Limitations on information-theoretically-secure quantum homomorphic encryption. Phys. Rev. A 90, 050303 (2014).

[b10] Childs A. M. Secure assisted quantum computation. Quantum Info. Comput. 5, 456–466 (2005).

[b11] Vu C. IBM makes quantum computing available on IBM cloud to accelerate innovation. IBM News Room http://www-03.ibm.com/press/us/en/pressrelease/49661.wss (2016).

[b12] Broadbent A., Fitzsimons J. & Kashefi E. in *Foundations of Computer Science, 2009. FOCS ‘09. 50th Annual* *IEEE Symposium* 517–526 (IEEE, 2009).

[b13] Briegel H., Browne D., Dür W., Raussendorf R. & Van den Nest M. Measurement-based quantum computation. Nat. Phys. 5, 19–26 (2009).

[b14] Morimae T. Continuous-variable blind quantum computation. Phys. Rev. Lett. 109, 230502 (2012). 23368174 10.1103/PhysRevLett.109.230502

[b15] Morimae T. & Fujii K. Blind topological measurement-based quantum computation. Nat. Commun. 3, 1036 (2012). 22948818 10.1038/ncomms2043 PMC3658012

[b16] Dunjko V., Kashefi E. & Leverrier A. Blind quantum computing with weak coherent pulses. Phys. Rev. Lett. 108, 200502 (2012). 23003133 10.1103/PhysRevLett.108.200502

[b17] Barz S. . Demonstration of blind quantum computing. Science 335, 303–308 (2012). 22267806 10.1126/science.1214707

[b18] Barz S., Fitzsimons J. F., Kashefi E. & Walther P. Experimental verification of quantum computation. Nat. Phys. 9, 727–731 (2013).

[b19] Giovannetti V., Maccone L., Morimae T. & Rudolph T. G. Efficient universal blind quantum computation. Phys. Rev. Lett. 111, 230501 (2013). 24476238 10.1103/PhysRevLett.111.230501

[b20] Sueki T., Koshiba T. & Morimae T. Ancilla-driven universal blind quantum computation. Phys. Rev. A 87, 060301 (2013).

[b21] Li Q., Chan W. H., Wu C. & Wen Z. Triple-server blind quantum computation using entanglement swapping. Phys. Rev. A 89, 040302 (2014).

[b22] Pérez-Delgado C. A. & Fitzsimons J. F. Iterated gate teleportation and blind quantum computation. Phys. Rev. Lett. 114, 220502 (2015). 26196609 10.1103/PhysRevLett.114.220502

[b23] Fisher K. . Quantum computing on encrypted data. Nat. Commun. 5, 3074 (2014). 24445949 10.1038/ncomms4074

[b24] Braunstein S. L. & van Loock P. Quantum information with continuous variables. Rev. Mod. Phys. 77, 513–577 (2005).

[b25] Weedbrook C. . Gaussian quantum information. Rev. Mod. Phys. 84, 621–669 (2012).

[b26] Holevo A. S., Sohma M. & Hirota O. Capacity of quantum gaussian channels. Phys. Rev. A 59, 1820–1828 (1999).

[b27] Marek P., Filip R. & Furusawa A. Deterministic implementation of weak quantum cubic nonlinearity. Phys. Rev. A 84, 053802 (2011).

[b28] Marshall K., Pooser R., Siopsis G. & Weedbrook C. Repeat-until-success cubic phase gate for universal continuous-variable quantum computation. Phys. Rev. A 91, 032321 (2015).

[b29] Menicucci N. C. . Universal quantum computation with continuous-variable cluster states. Phys. Rev. Lett. 97, 110501 (2006). 17025869 10.1103/PhysRevLett.97.110501

[b30] Braunstein S. L. & Kimble H. J. Teleportation of continuous quantum variables. Phys. Rev. Lett. 80, 869–872 (1998).

[b31] Miyata K. . Experimental realization of a dynamic squeezing gate. Phys. Rev. A 90, 060302(R) (2014).

[b32] Pirandola S., Eisert J., Weedbrook C., Furusawa A. & Braunstein S. L. Advances in quantum teleportation. Nat. Photon. 9, 641–652 (2015).

[b33] Andersen U. L. & Ralph T. C. High-fidelity teleportation of continuous-variable quantum states using delocalized single photons. Phys. Rev. Lett. 111, 050504 (2013). 23952378 10.1103/PhysRevLett.111.050504

[b34] Sangouard N., Simon C., de Riedmatten H. & Gisin N. Quantum repeaters based on atomic ensembles and linear optics. Rev. Mod. Phys. 83, 33–80 (2011).

[b35] Hansch T. W. & Couillaud B. Laser frequency stabilization by polarization spectroscopy of a reflecting reference cavity. Opt. Commun. 35, 441–444 (1980).

[b36] Lvovsky A. I. & Raymer M. G. Continuous-variable optical quantum-state tomography. Rev. Mod. Phys. 81, 299–332 (2009).

