# Supplementary material for: Continuous-variable quantum computing on encrypted data
Source: Nat Commun. 2016 Dec 14;7:13795. doi: 10.1038/ncomms13795 (PMC5171809; doi:10.1038/ncomms13795)
Supplement: Supplementary Information — Supplementary Figures 1-9, Supplementary Table 1, Supplementary Notes 1-8, Supplementary Methods and Supplementary References. [file ncomms13795-s1.pdf]

**SUPPLEMENTARY FIGURES**

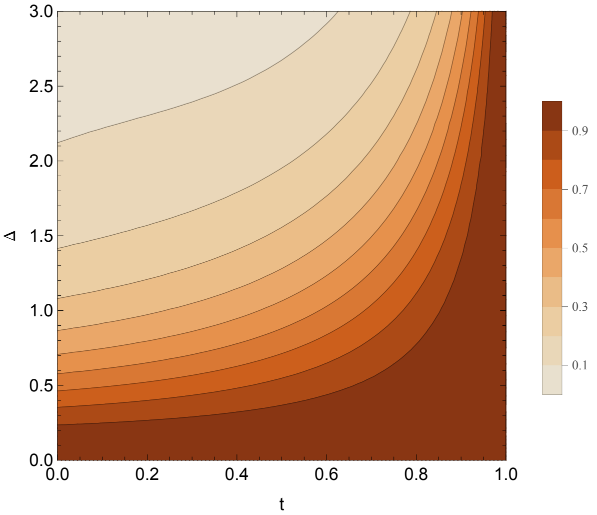


Supplementary Figure 1: Average fidelity between $|\langle\alpha+\beta|t^2\alpha+t\beta\rangle|^2$ for an initial coherent state subject to the protocol with and without loss, which corresponds to a channel with transmission factor $t$. Both $\alpha$ and $\beta$ are distributed according to Gaussians with zero mean and variance $\Delta^2$.

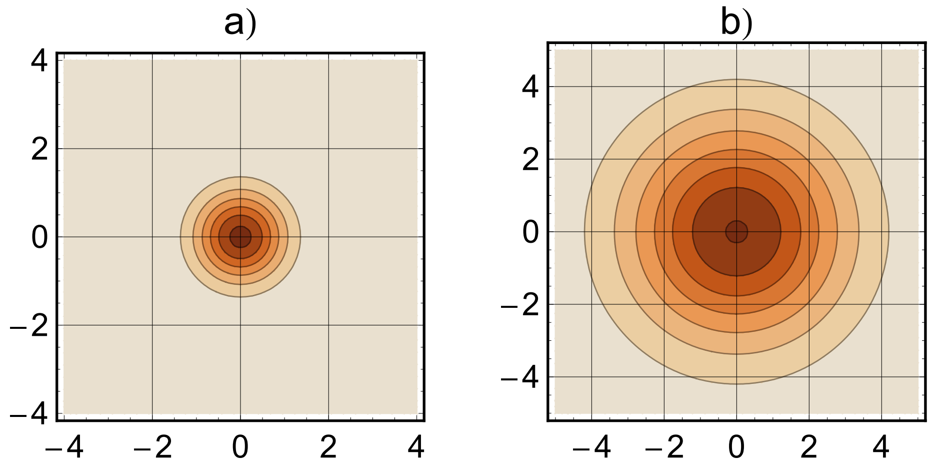


Supplementary Figure 2: (a) The Wigner function corresponding to the vacuum state. (b) The Wigner function after the vacuum is encrypted where $\Delta = 2$; this corresponds to a thermal state with $\bar{n} = 8$.

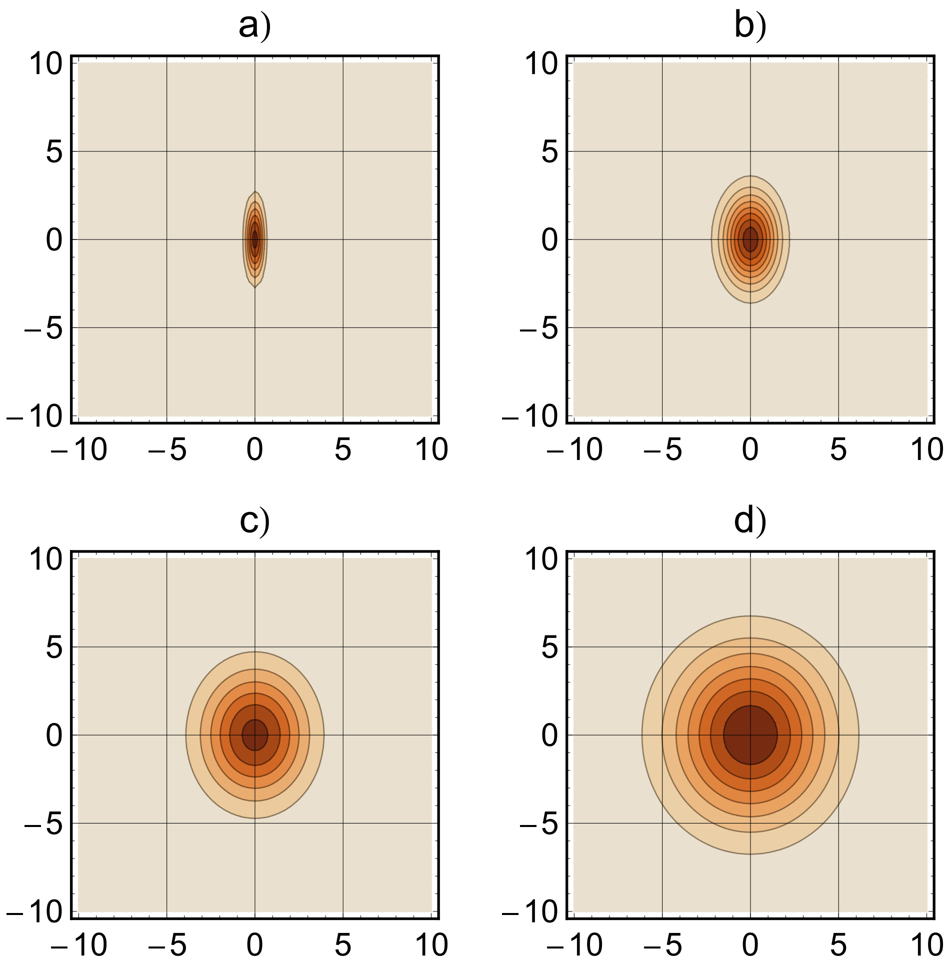


Supplementary Figure 3: (a) The Wigner function for a squeezed vacuum state with squeezing parameter $r = \ln 2$. (b,c,d) The Wigner function after the squeezed state is encrypted with parameter $\Delta = 1, 2, 3$ respectively. Notice that for small $\Delta$ the encrypted state is asymmetric, which gives us some information about the initial state, but for higher values the plot more closely resembles a thermal state. The purity of the resulting states (a-d) are $\approx 1, 0.27, 0.10, 0.05$ respectively.

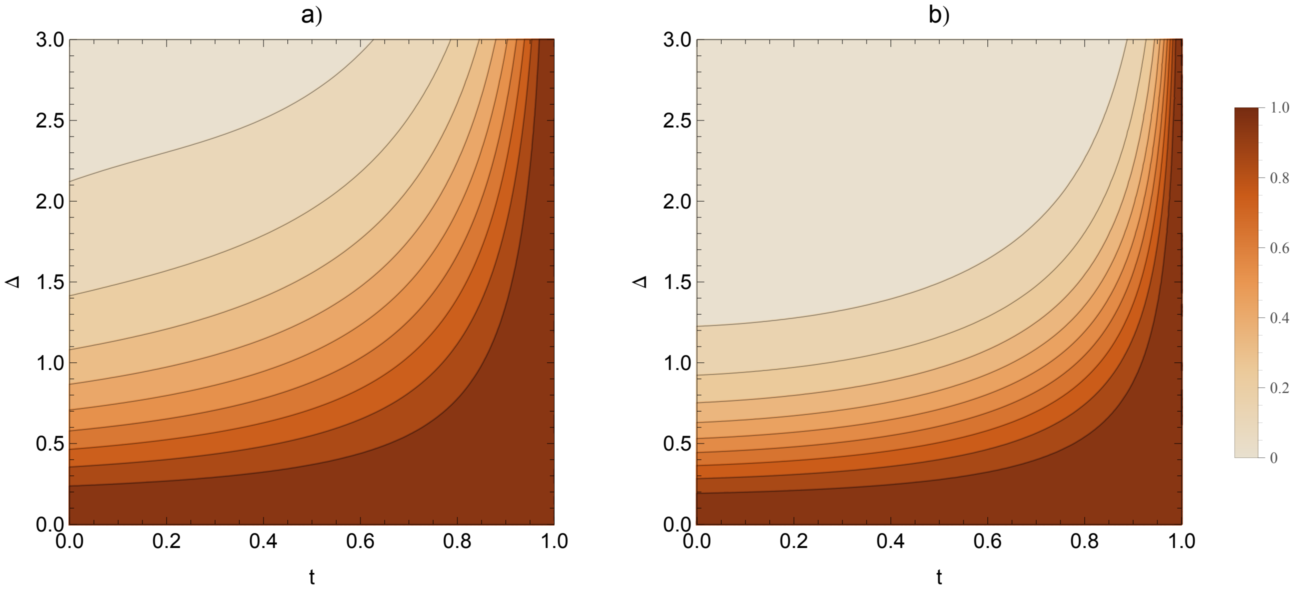


Supplementary Figure 4: (a) Average fidelity between $|\langle\alpha+\beta|t^2\alpha+t\beta\rangle|^2$ for an initial coherent state subject to the protocol with and without loss, which corresponds to a channel with transmission factor $t$. (b) The same plot as in part (a) but where the final displacement does not take into account the channel loss $t$, given by $|\langle\alpha+\beta|t^2\alpha+t\beta\rangle+(t^2-1)\gamma|^2$, where $\gamma$ is the encryption parameter. All of $\alpha, \beta$ and $\gamma$ are distributed according to Gaussians with zero mean and variance $\Delta^2$.

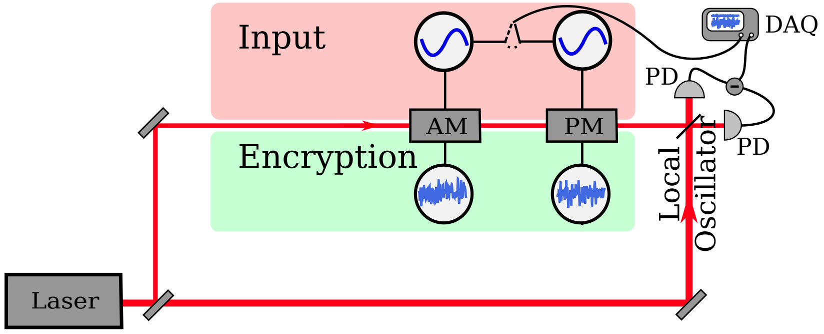


Supplementary Figure 5: A schematic representation of the encryption efficiency experiment. PD: Photo diode. DAQ: Data acquisition.

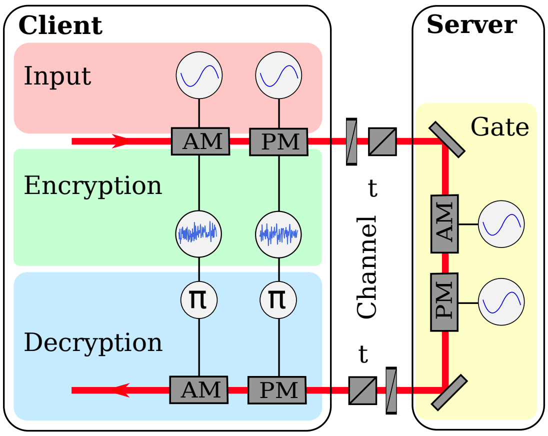


Supplementary Figure 6: A schematic representation of the displacement gate experiment.

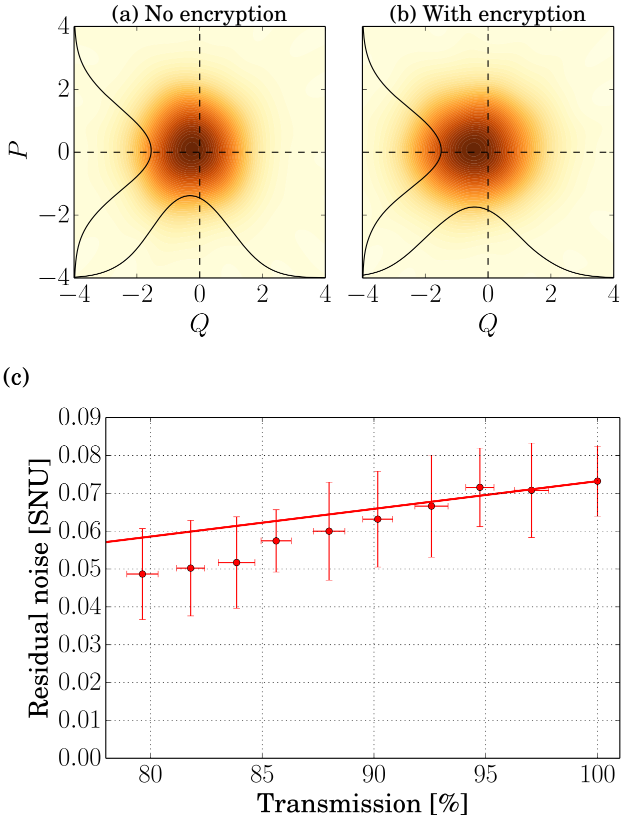


Supplementary Figure 7: **The effect of encryption and decryption on the computed data.** (a,b) Reconstructed Wigner functions of the ensembles of coherent states representing the output states, i.e. the input state ($V_{\mathrm{in}} = 0.3$ SNU) after the gate operation ($V_{\mathrm{gate}} = 0.6$ SNU). (a) Without encrypting the data and (b) with encryption ($V_{\mathrm{enc}} = 31$ SNU). (c) Plot of the residual noise of the decryption process as a function of transmission. The solid line is a theory curve of the residual noise versus transmission calculated by starting with the measured residual noise value of the ideal channel ($T = 100\,\%$). The data was recorded with a spectrum analyser measuring zero-span around 10.5 MHz with a resolution bandwidth of 300 kHz and a video bandwidth of 30 Hz. The size of the error bars is due to the standard deviation of the 1000 recorded points.

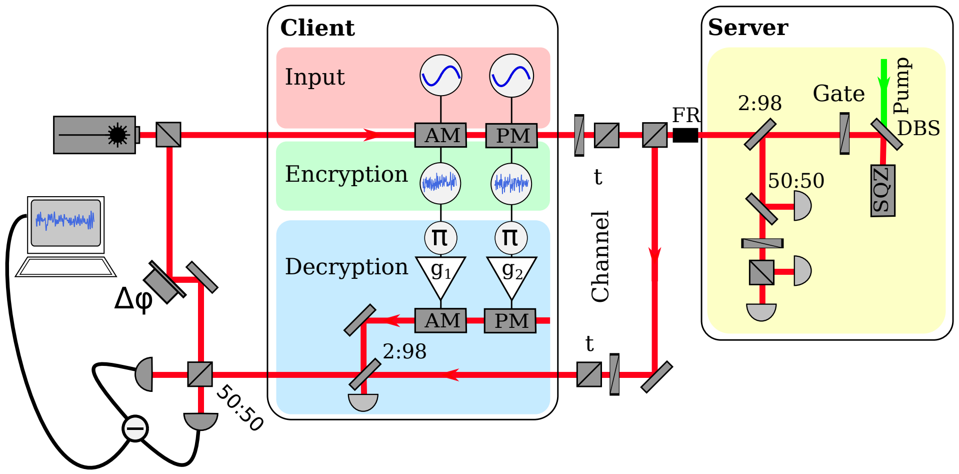


Supplementary Figure 8: A schematic representation of the squeezing gate experiment.

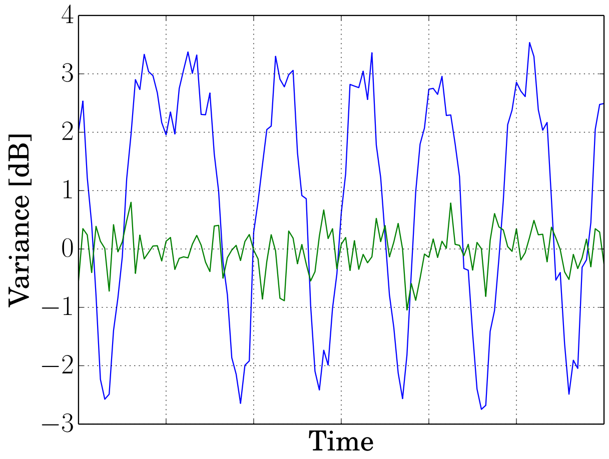


Supplementary Figure 9: Variance of the squeezed state used for the quantum gate, with a scanned local oscillator. The green trace represents shotnoise.

## SUPPLEMENTARY TABLES

| Step | Operation | Transformation |
|---|---|---|
| Initial | | $W(q_1, p_1)$ |
| Encryption | $D_1(Q, P)$ | $W(q_1 - Q, p_1 - P)$ |
| Loss | | $W(q_1 - Q, p_1 - P)W_{vac}(q_2, p_2)$ |
| | $B_{12}(t)$ | $W(t(q_1 - Q) + rq_2, t(p_1 - P) + rp_2)\times$ |
| | | $W_{vac}(-r(q_1 - Q) + tq_2, -r(p_1 - P) + tp_2)$ |
| Server | $D_1(A, B)$ | $W(q_1' - A, p_1' - B)W_{vac}(q_2', p_2')$ |
| Loss | | $W(q_1' - A, p_1' - B)W_{vac}(q_2', p_2')W_{vac}(q_3, p_3)$ |
| | $B_{13}(t)$ | $W(t(q_1' - A) + rq_3, t(p_1' - B) + rp_3)W_{vac}(q_2', p_2')W_{vac}(-r(q_1' - A) + tq_3, -r(p_1 - A') + tp_3)$ |
| Decryption | $D_1(-t^2Q, -t^2P)$ | $W(t^2q_1 - tA + trq_2 + rq_3, t^2p_1 - tB + trp_2 + rp_3)W_{vac}(q_2', p_2')W_{vac}(q_3', p_3')$ |

Supplementary Table 1: The transformations induced by the protocol in the framework of the Wigner function, where we have limited the server to simply applying a displacement. The calculation for $U_2(T)$ proceeds in a similar fashion, but $U_3(T)$ does not have a linear transformation in terms of the operators $\hat{q}, \hat{p}$ and would be more complicated to calculate in this fashion.

## SUPPLEMENTARY NOTES

### Supplementary Note 1: Gate Decryption Operators

In this section we first define a set of continuous-variable gates as well as the following conventions.

$$
\begin{aligned}
[\hat{q}, \hat{p}] &= i \\
X(Q) &= \exp(-iQ\hat{p}) \\
Z(P) &= \exp(iP\hat{q}) \\
U_k(T) &= \exp(iT\hat{q}^k) \\
F &= \exp\left[\frac{i\pi}{4}\left(\hat{q}^2 + \hat{p}^2\right)\right] \\
C_{Z,12} &= \exp(i\hat{q}_1 \otimes \hat{q}_2) \\
D(\alpha) &= \exp(\alpha\hat{a}^\dagger - \alpha^*\hat{a})
\end{aligned}
$$

$$\begin{aligned} D(Q,P) &= e^{-\frac{i}{2}QP} Z(P)X(Q) \text{ for } \alpha = \frac{1}{\sqrt{2}}(Q+iP) \\ |\psi\rangle_L &= D(Q,P)|\psi\rangle \end{aligned} \tag{1}$$

Identities:

$$\begin{aligned} e^X e^Y &= \exp\{Y + \sum_{n=1}^{\infty} \frac{1}{n!}[X,Y]_{(n)}\} e^X \\ [X,Y]_{(n)} &= [X,[X,[X,\ldots,[X,Y\underbrace{],\ldots,]]]}_{n-\text{brackets}} \end{aligned} \tag{2}$$

We define a encryption operation, $D(Q,P)$, consisting of a random displacement in phase space. We consider a universal set of gates $G \in \{X(Q), Z(P), U_2(T), U_3(T), F, C_{Z,12}\}$. We wish to demonstrate the existence of a correction operator, $C(Q,P,G)$, which depends on the encryption parameters and $G$ such that $C(Q,P,G)GD(Q,P)|\psi\rangle = G|\psi\rangle$.

$Z(S)$

$$\begin{aligned} X(Q)Z(S)X^\dagger(Q) &= \exp(-iQ\hat{p})\exp(iS\hat{q})\exp(iQ\hat{p}) \\ &= \exp(iS\hat{q} + [-iQ\hat{p}, iS\hat{q}] + \ldots) \\ &= \exp(iS\hat{q} - iQS) \\ \Rightarrow X(Q)Z(S) &= e^{-iQS}Z(S)X(Q) \end{aligned} \tag{3}$$

Correction:

$$\begin{aligned} Z(S)D(Q,P) &= Z(S)e^{-\frac{i}{2}QP}Z(P)X(Q) \\ &= e^{-\frac{i}{2}QP}Z(P)Z(S)X(Q) \\ &= e^{-\frac{i}{2}QP}Z(P)e^{iQS}X(Q)Z(S) \\ &= C^\dagger(Q,P,S)Z(S) \\ C(Q,P,S) &= \exp[i(QP/2 - QS)]X(-Q)Z(-P) \end{aligned} \tag{4}$$

$X(S)$

Correction:

$$\begin{aligned} X(S)D(Q,P) &= X(S)e^{-\frac{i}{2}QP}Z(P)X(Q) \\ &= e^{-\frac{i}{2}QP}e^{-iPS}Z(P)X(S)X(Q) \quad \text{By (3)} \\ &= C^\dagger(Q,P,S)X(S) \\ C(Q,P,S) &= \exp[i(QP/2 + PS)]X(-Q)Z(-P) \end{aligned} \tag{5}$$

$U_2(T)$

$$\begin{aligned} X(Q)U_2(T)X^\dagger(Q) &= \exp(-iQ\hat{p})\exp(iT\hat{q}^2)\exp(iQ\hat{p}) \\ &= \exp(iT\hat{q}^2 + [-iQ\hat{p}, iT\hat{q}^2] + \ldots) \\ &= \exp(iT\hat{q}^2 - 2iQT\hat{q} + \frac{1}{2!}[-iQ\hat{p}, -2iQT\hat{q}] + \ldots) \\ &= \exp(iT\hat{q}^2 - 2iQT\hat{q} + \frac{1}{2}(2iQ^2T) + \ldots) \end{aligned}$$

$$
\begin{aligned}
&= \exp(iT\hat{q}^2 - 2iQT\hat{q} + iQ^2T) \\
\Rightarrow X(Q)U_2(T) &= \exp(iT\hat{q}^2 - 2iQT\hat{q} + iQ^2T)X(Q) \\
&= e^{iQ^2T}Z(-2QT)U_2(T)X(Q) \qquad (6)
\end{aligned}
$$

Correction:

$$
\begin{aligned}
U_2(T)D(Q,P) &= U_2(T)e^{-\frac{i}{2}QP}Z(P)X(Q) \\
&= e^{-\frac{i}{2}QP}Z(P)U_2(T)X(Q) \\
&= e^{-\frac{i}{2}QP}Z(P)e^{-iQ^2T}Z(2QT)X(Q)U_2(T) \\
&= C^\dagger(Q,P,T)U_2(T) \\
\Rightarrow C(Q,P,T) &= \exp[i(QP/2 + Q^2T)]X(-Q)Z(-2QT)Z(-P) \qquad (7)
\end{aligned}
$$

$U_3(T)$

$$
\begin{aligned}
X(Q)U_3(T)X^\dagger(Q) &= \exp(-iQ\hat{p})\exp(iT\hat{q}^3)\exp(iQ\hat{p}) \\
&= \exp(iT\hat{q}^3 + [-iQ\hat{p}, iT\hat{q}^3] + \ldots) \\
&= \exp(iT\hat{q}^3 - 3iQT\hat{q}^2 + \frac{1}{2!}[-iQ\hat{p}, -3iQT\hat{q}^2] + \ldots) \\
&= \exp(iT\hat{q}^3 - 3iQT\hat{q}^2 + \frac{1}{2!}(6iQ^2T\hat{q}) + \frac{1}{3!}[-iQ\hat{p}, 6iQ^2T\hat{q}] + \ldots) \\
&= \exp(iT\hat{q}^3 - 3iQT\hat{q}^2 + 3iQ^2T\hat{q} + \frac{1}{6}(-6iQ^3T) + \ldots) \\
&= \exp(iT\hat{q}^3 - 3iQT\hat{q}^2 + 3iQ^2T\hat{q} - iQ^3T) \\
\Rightarrow X(Q)U_3(T) &= e^{-iQ^3T}Z(3Q^2T)U_2(-3QT)U_3(T)X(Q) \qquad (8)
\end{aligned}
$$

Correction:

$$
\begin{aligned}
U_3(T)D(Q,P) &= U_3(T)e^{-\frac{i}{2}QP}Z(P)X(Q) \\
&= e^{-\frac{i}{2}QP}Z(P)U_3(T)X(Q) \\
&= e^{-\frac{i}{2}QP}Z(P)e^{iQ^3T}U_2(3QT)Z(-3Q^2T)X(Q)U_3(T) \\
&= C^\dagger(Q,P,T)U_3(T) \\
C(Q,P,T) &= \exp[i(QP/2 - Q^3T)]X(-Q)Z(3Q^2T)U_2(-3QT)Z(-P) \qquad (9)
\end{aligned}
$$

However, $U_2(T)$ does not slide nicely through $F$ so we wish to have the server handle this correction on the fly.

$$
U_3(T)D(Q,P) = \exp[i(-QP/2 + Q^3T)]U_2(3QT)Z(P - 3Q^2T)X(Q)U_3(T) \qquad (10)
$$

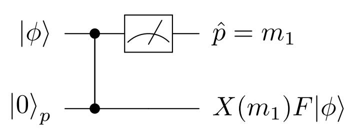


(11)

Let $|\phi\rangle = F^\dagger U_3(T)D(Q,P)|\psi\rangle$ so that the output of the teleportation is given by

$$
\begin{aligned}
&X(m_1)U_3(T)D(Q,P)|\psi\rangle \\
&= \exp[i(-QP/2 + Q^3T)]X(m_1)U_2(3QT)Z(P - 3Q^2T)X(Q)U_3(T)|\psi\rangle. \qquad (12)
\end{aligned}
$$

It is clear that applying the correction operator $U_2(-3QT)X(-m_1)$ to this state would eliminate the appearance of $U_2$, however we cannot divulge the value of $Q$ as this would compromise the security. Consider the circuit given by

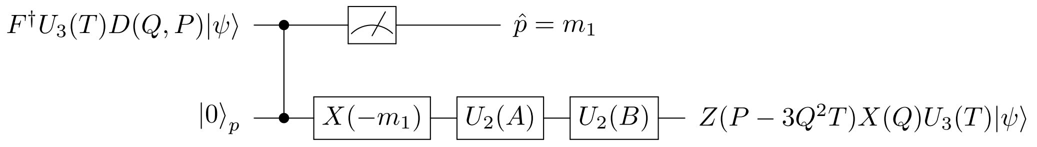

(13)

where $A+B=-3QT$. We can then slide $U_2(A)$ all the way to the left as

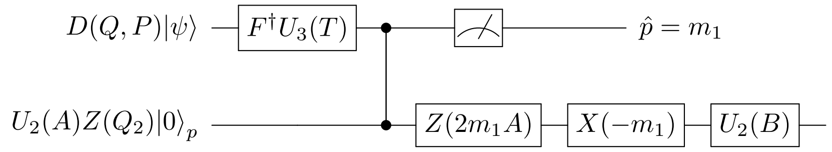

(14)

where the output is given by $Z(Q_2)Z(2m_1A)Z(P-3Q^2T)X(Q)U_3(T)|\psi\rangle$ and we also include another $Z$ gate as part of the preparation. Note that the $Z(2m_1A)$ gate can be deferred to the client, who knows the value of $A$, by including it as an update to their decryption key. If we choose $A$ at 'random' then we are giving the server negligible information about the encryption parameter $Q$ by telling them the value of $B$. In particular, we can see the transmission of $-3QT$ is modulated by the Gaussian noise $A$ and this channel behaves as an additive white Gaussian noise channel. Explicitly, the channel capacity goes as $C=\log_2(1+9T^2\mathrm{Var}(Q)/\mathrm{Var}(A))$, and so for large enough displacements, $A$, this can be made negligible. Note that the server will also have to inform the client of $m_1$ obtained so that they can correct for the additional $Z(2m_1A)$ gate, and thus implementing this gate requires a single round of communication. Furthermore the density operator corresponding to the state $U_2(A)Z(Q_2)|0\rangle_p$ when averaged over $Q_2$ is proportional to the identity, and thus no information about $Q$ can be attained from the state itself. This can be seen from the fact that

$$\begin{aligned}\int dQ_2\; P(Q_2)\; Z(Q_2)|0\rangle_p\langle 0|_p Z^\dagger(Q_2) &= \int dQ_2\; P(Q_2)\; |Q_2\rangle_p\langle Q_2|_p \\ &\to \int dQ_2\; |Q_2\rangle_p\langle Q_2|_p \propto \mathbb{1}, \end{aligned} \tag{15}$$

if we choose $Q_2$ according to some Gaussian probability density function, $P(Q_2)$, whose variance tends towards infinity.

$C_{Z,12}$

$$\begin{aligned} C_{Z,12}[X(Q)\otimes\mathbb{1}]C^\dagger_{Z,12} &= \exp(i\hat{q}_1\otimes\hat{q}_2)[\exp(-iQ\hat{p}_1)\otimes\mathbb{1}]\exp(-i\hat{q}_1\otimes\hat{q}_2) \\ &= \exp(i\hat{q}_1\hat{q}_2)[\exp(-iQ\hat{p}_1)]\exp(-i\hat{q}_1\hat{q}_2) \quad \text{Tensor structure implicit in subscripts} \\ &= \exp(-iQ\hat{p}_1+[i\hat{q}_1\hat{q}_2,-iQ\hat{p}_1]+\ldots) \\ &= \exp(-iQ\hat{p}_1+iQ\hat{q}_2) \\ &= X_1(Q)Z_2(Q) \end{aligned} \tag{16}$$

$$\begin{aligned} C_{Z,12}[X_1(Q_1)\otimes X_2(Q_2)]C^\dagger_{Z,12} &= C_{Z,12}[(X_1(Q_1)\otimes\mathbb{1}_2)(\mathbb{1}_1\otimes X_2(Q_2)]C^\dagger_{Z,12} \\ &= C_{Z,12}[(X_1(Q_1)\otimes\mathbb{1}_2)C^\dagger_{Z,12}C_{Z,12}(\mathbb{1}_1\otimes X_2(Q_2)]C^\dagger_{Z,12} \\ &= [X_1(Q_1)\otimes Z_2(Q_1)][Z_1(Q_2)\otimes X_2(Q_2)] \\ &= X_1(Q_1)Z_1(Q_2)\otimes Z_2(Q_1)X_2(Q_2) \\ \Rightarrow C_{Z,12}[X_1(Q_1)\otimes X_2(Q_2)] &= [X_1(Q_1)Z_1(Q_2)\otimes Z_2(Q_1)X_2(Q_2)]C_{Z,12} \end{aligned} \tag{17}$$

Correction:

$$\begin{aligned} C_{Z,12}[D_1(Q_1,P_1)\otimes D_2(Q_2,P_2)] &= C_{Z,12}e^{-\frac{i}{2}(Q_1P_1+Q_2P_2)}[Z_1(P_1)X_1(Q_1)\otimes Z_2(P_2)X_2(Q_2)] \\ &= e^{-\frac{i}{2}(Q_1P_1+Q_2P_2)}[Z_1(P_1)\otimes Z_2(P_2)]C_{Z,12}[X_1(Q_1)\otimes X_2(Q_2)] \end{aligned}$$

$$
\begin{aligned}
&= e^{-\frac{i}{2}(Q_1P_1+Q_2P_2)}[Z_1(P_1)\otimes Z_2(P_2)][X_1(Q_1)Z_1(Q_2)\otimes Z_2(Q_1)X_2(Q_2)]C_{Z,12} \\
&= e^{-\frac{i}{2}(Q_1P_1+Q_2P_2)}[Z_1(P_1)X_1(Q_1)Z_1(Q_2)\otimes Z_2(P_2)Z_2(Q_1)X_2(Q_2)]C_{Z,12} \\
&= C^\dagger(Q_1,P_1,Q_2,P_2)C_{Z,12} \\
C(Q_1,P_1,Q_2,P_2) &= e^{\frac{i}{2}(Q_1P_1+Q_2P_2)}[Z_1(-Q_2)X_1(-Q_1)Z_1(-P_1)\otimes X_2(-Q_2)Z_2(-Q_1)Z_2(-P_2)] \qquad (18)
\end{aligned}
$$

$F$

$$
\begin{aligned}
FZ(P)F^\dagger &= X(-P) \\
FX(Q)F^\dagger &= Z(Q) \\
FD(Q,P) &= Fe^{-\frac{i}{2}QP}Z(P)X(Q) \\
&= e^{-\frac{i}{2}QP}FZ(P)F^\dagger FX(Q)F^\dagger F \\
&= e^{-\frac{i}{2}QP}X(-P)Z(Q)F \\
&= C^\dagger(Q,P)F \\
C(Q,P) &= e^{\frac{i}{2}QP}Z(-Q)X(P) \qquad (19)
\end{aligned}
$$

**Supplementary Note 2: Compositions of Gates**

To implement a single gate from the universal set defined above, we have demonstrated that there exists a correction (decryption) operator, $C_G$, that satisfies

$$\hat{C}_G\hat{G}\hat{D}(Q,P)|\psi\rangle = \hat{G}|\psi\rangle, \qquad (20)$$

and similarly for the two-qumode $C_{Z,12}$ gate. We would like the server to be able to compose gates without needing the client to decrypt (or correct) between applications. Namely we wish to demonstrate that

$$\hat{C}'\hat{G}_2\hat{G}_1\hat{D}(Q,P)|\psi\rangle = \hat{G}_2\hat{G}_1|\psi\rangle, \qquad (21)$$

or that the client can update their correction operator while only requiring possibly classical communication with the server. Suppose that

$$
\begin{aligned}
\hat{C}_1\hat{G}_1\hat{D}(Q,P)|\psi\rangle &= \hat{G}_1|\psi\rangle \\
\hat{C}_2\hat{G}_2\hat{D}(Q,P)|\psi\rangle &= \hat{G}_2|\psi\rangle, \qquad (22)
\end{aligned}
$$

can we construct a $\hat{C}'$ from our knowledge of $\hat{C}_{1,2}$? Notice that for all of the gates in the universal set the decryption operators, $C$, involve (up to a phase) only products of operators from the set $C \in \{X(Q), Z(P)\}$; aside from the special case of $U_3(T)$ discussed above. Suppose $G \in \{X(Q), Z(P), U_2(T), U_3(T), F, C_{Z,12}\}$ we would like to show that there exists a modified decryption operator $C'$ such that

$$C'G = GD(Q,P)C \qquad (23)$$

so that we can *slide* a new correction through the gate as

$$
\begin{aligned}
C_1'G_2G_1D(Q,P)|\psi\rangle &= [G_2D(Q,P)C_1]G_1D(Q,P)|\psi\rangle \\
&= G_2D(Q,P)[C_1G_1D(Q,P)]|\psi\rangle \\
&= G_2D(Q,P)G_1|\psi\rangle \\
\Rightarrow C_2C_1'G_2G_1D(Q,P)|\psi\rangle &= C_2G_2D(Q,P)G_1|\psi\rangle \\
&= G_2G_1|\psi\rangle. \qquad (24)
\end{aligned}
$$

Notice first that gates, $G$, with Hamiltonians involving only powers of $\hat{q}$ slide trivially through each other. The only correction operator, in $\{X(Q), Z(P)\}$, that does not involve powers of only $\hat{q}$ is precisely $X(Q)$. But we have already shown how to rewrite

$$C'G = GX(Q), \tag{25}$$

it is easy to insert the extra displacement $D(Q, P)$ between the two terms in the RHS of the above equation since $Z(P), X(Q)$ easily slide through all elements $G$ as illustrated in the above work. Also note for the case where $G = X(Q)$ and $C$ involving powers of $\hat{q}$ we can apply the same identities by shifting the extra terms to the other side. By recursively applying the identities to swap orderings by applying new corrections we can not only slide the *gate* through the *encryption* operation, we can also slide *corrections* through other *gates*. In this manner we can build up more complicated gates, with no communication between the client and server for Gaussian gates and one additional round of communication for $U_3$.

### Supplementary Note 3: Effect of Transmission

To consider the action of each step of the protocol on our state we move to the Wigner function representation; this is convenient as all operations of interest are linear transformations in the canonical operations $\hat{q}, \hat{p}$ and thus are simple transformations of the associated Wigner function. We model the loss as a beamsplitter $B_{12}(t)$

$$B_{12}(t) = \begin{pmatrix} \cos\theta & \sin\theta \\ -\sin\theta & \cos\theta \end{pmatrix}, \tag{26}$$

where the transmission (reflection) factor is given by $t = \cos\theta$ ($r = \sin\theta$). This is associated with the input-output relations

$$\begin{pmatrix} q_1' \\ q_2' \end{pmatrix} = B_{12}(t) \begin{pmatrix} q_1 \\ q_2 \end{pmatrix}, \tag{27}$$

and similarly for $p_{1,2}$. When subjecting our mode of interest to loss we inject the vacuum state, $W_{vac}(q, p) = 1/\pi \exp(-q^2 - p^2)$, in the spare port of the beam splitter. We can study the effect of loss in comparison to the ideal case by integrating over the loss modes and calculating the fidelity between the resulting Wigner function and the one that would be obtained in the absence of loss. If the initial state is a coherent state, $|\alpha\rangle$, then these steps lead to the transformation $|\alpha\rangle \to |t^2\alpha + t\beta\rangle$ where $\beta$ is the displacement provided by the server, the general case is presented in Supplementary Table 1. The symmetric case, where the encryption and the server's displacement are distributed according to a Gaussian probability distribution function with zero mean and variance $\Delta^2$, is plotted in Supplementary Fig. 1.

### Supplementary Note 4: Imperfect Encryption

To encrypt a state we used the fact that randomly shifting the initial state in phase space results in the maximally mixed state

$$X(|\psi\rangle\langle\psi|) = \frac{1}{\pi} \int D(\alpha)|\psi\rangle\langle\psi|D^\dagger(\alpha) d^2\alpha = \mathbb{1}. \tag{28}$$

To see why this is true, notice that $D(\beta)$ provides an irreducible representation, up to a phase, of the Heisenberg-Weyl group. Furthermore, the operator $X$ commutes with $D(\beta)$ and hence by Schur's Lemma, for unitary groups, $X \propto \mathbb{1}$. Using the fact that $|\psi\rangle$ is normalized it is easy to show that the constant of proportionality is 1, and thus we have the relationship. From a physical point of view it is not possible to apply a random displacement over $\mathbb{R}^2$ as this would require infinite energy; from a mathematical perspective it's not clear what a 'random' displacement over $\mathbb{R}^2$ even means. Instead we consider applying a displacement that is Gaussian distributed with zero mean and variance $\Delta^2$ which follows the mapping

$$X(|\psi\rangle\langle\psi|) = \frac{1}{2\pi\Delta^2} \int D(\alpha)|\psi\rangle\langle\psi|D^\dagger(\alpha) e^{-\frac{|\alpha|^2}{2\Delta^2}} d^2\alpha. \tag{29}$$

The finite width of the Gaussian will lead to this state differing from the maximally mixed state; in general the resulting state will depend on the initial state. A simple figure of merit we can study is the purity of the state $\mathrm{Tr}\rho^2$, in discrete variable systems the purity is always greater than $1/d$ but in continuous variable systems the purity can be zero, as is the case for the maximally

mixed state. We can find the purity of an arbitrary initial state, $\rho \to W(\alpha)$, subject to the map $X(|\psi\rangle\langle\psi|)$ by working in the Wigner function formalism

$$W(\alpha) \to W(\alpha') = \frac{1}{2\pi\Delta^2}\int W(\alpha-\beta)e^{-\frac{|\beta|^2}{2\Delta^2}}d^2\beta$$
$$\mathrm{Tr}\rho^2 = 2\pi\int W(\alpha')^2 d^2\alpha'. \tag{30}$$

Note that this transformation is also just the convolution of our initial Wigner function with a Gaussian probability distribution. We can then use the purity to characterize how well our encryption operation simulates the ideal encryption procedure, given a particular class of input states. Consider the case where our initial state is also a coherent state, given by $|\alpha\rangle$

$$\begin{aligned} X(|\alpha\rangle\langle\alpha|) &= \frac{1}{2\pi\Delta^2}\int D(\beta)|\alpha\rangle\langle\alpha|D^\dagger(\beta)e^{-\frac{|\beta|^2}{2\Delta^2}}d^2\beta \\ &= \frac{1}{2\pi\Delta^2}\int |\alpha+\beta\rangle\langle\alpha+\beta|e^{-\frac{|\beta|^2}{2\Delta^2}}d^2\beta \\ &= \frac{1}{2\pi\Delta^2}\int |\gamma\rangle\langle\gamma|e^{-\frac{|\gamma-\alpha|^2}{2\Delta^2}}d^2\gamma. \end{aligned} \tag{31}$$

Note that a thermal state $\rho_{th}(\bar{n})$ of average excitation number $\bar{n}$ has Glauber-Sudarshan representation given by

$$\rho_{th}(\bar{n}) = \frac{1}{\pi\bar{n}}\int |\alpha\rangle\langle\alpha|e^{-\frac{|\alpha|^2}{\bar{n}}}d^2\alpha. \tag{32}$$

Thus we have that $X(|\alpha\rangle\langle\alpha|)$ corresponds to a thermal state with $\bar{n} = 2\Delta^2$ which is displaced by an amount $\alpha$. For a fixed amount of energy, thermal states maximize their Von Neumann entropy $S(\rho) = -\mathrm{Tr}(\rho\log\rho)$ which is also related to purity; $S(\rho)$ is maximized by the maximally mixed state and is zero for pure states. The purity of $\rho_{th}(\bar{n})$ can be calculated as $\mathrm{Tr}(\rho_{th}(\bar{n})^2) = 1/(1+2\bar{n})$ so that the purity of $X(|\alpha\rangle\langle\alpha|)$ is given by $1/(1+4\Delta^2)$.

In general one can use the Wigner function to evaluate the action of the encryption and to determine how mixed the resulting state is. In Supplementary Fig. 2 we show the action of imperfect encryption on the vacuum state and in Supplementary Fig. 3 we show the same for a squeezed vacuum state. In general, we want the Gaussian envelope to be large enough to smear out any details about the set of possible initial states.

### Supplementary Note 5: Entanglement-Based Analogy

One can exploit the well-known equivalence between preparing a coherent state, chosen according to a Gaussian distribution, and performing Heterodyne detection on half of an EPR pair to show an analogous entanglement-based encryption scheme. In conventional CV teleportation [1], where the parties share a perfectly squeezed EPR pair, the final correction operator is precisely a displacement. Using the principle of deferred measurement, where we simply wait to perform the two homodyne measurements until after the state has been returned from the server, it is clear that the server could not have known of the measurement outcomes and hence the encryption parameters. This analogy works only in the limit of infinite squeezing; in the realistic scenario of finite squeezing there is excess noise introduced onto the mode of interest and the entanglement-based scheme is not a perfect analogy. However, in the limit of large squeezing, or equivalently a Gaussian distribution with large variance, the excess noise tends to zero and this is a good approximation to the actual implementation.

One can also show that the interactive $U_3(T)$ gate has an entanglement-based equivalent. First note that,

$$\begin{aligned} U_{2,b}(A)|EPR\rangle &\propto U_{2,b}(A)\int dq|q\rangle_a|q\rangle_b \\ &\propto \int dq e^{iAq^2}\int dp e^{-iqp}|q\rangle_a|p\rangle_b. \end{aligned} \tag{33}$$

If we now measure the second mode and obtain an outcome $P$ we have the following,

$$\begin{aligned} &\to \int dq e^{iAq^2}e^{-iqP}|q\rangle_a \\ &= U_{2,a}(A)\int dq e^{-iqP}|q\rangle_a \end{aligned}$$

$$\propto U_2(A)Z(-P)|0\rangle_p. \tag{34}$$

Thus we can use the above procedure to modify the implementation of $U_3(T)$ and defer the final measurement to construct an entanglement-based version. Explicitly, the client can implement $U_2(A)$ on half of an EPR pair, shared with the server, before measuring the $\hat{p}$ quadrature; this will have the same effect as the previous scheme. As before, this holds in an exact sense only in the unphysical limit of perfect squeezing.

## Supplementary Note 6: Channel Estimation

When accounting for loss, as seen in 1, we require the displacement $D(-t^2Q, -t^2P)$ as part of the correction. To perform this displacement we must know the transmission coefficient $t$, however we could forgo this stipulation and assume that $t \approx 1$, but doing so will result in a reduced fidelity. In order to partially correct for loss it is necessary to do parameter estimation on the channel. This can be done, for example, by periodically sending random coherent states and asking the server to apply some random displacement, after which one can measure the state in order to learn information about the loss parameter. Generally, there will also be additional noise that one can not correct for as well, but if desired one can estimate the magnitude of this noise by sending an appropriate ensemble of initial states. By doing this frequently enough one can ensure that the channel is not varying appreciably with time, and if so to adjust the necessary correction.

This process ensures that the server is not maliciously altering the channel parameters, in fact we can take this notion one step further and check that the server is indeed performing the desired displacement thus verifying the $X, Z$ operations in our full calculation. In the original proposal for blind quantum computation, the ability to verify that the server is performing the desired calculation arises from the capability of hiding the gates that one is performing. Through doing so one is able to periodically perform a check by doing an easy computation of which one already knows the answer. Since the server is unable to discriminate this check from the actual computation it is unable to pass the check without operating faithfully. In our case the server is fully aware of all gates being performed and could for example perform all operations except $U_3(T)$ as intended. Fortunately, there is a large class of desirable computations for which there exist efficient classical verification methods and so one is able to detect an incorrect answer with classical post-processing.

We show the advantage of accounting for the loss in the channel by comparing the fidelities to the ideal state both with and without taking into account the loss parameter in Supplementary Fig. 4.

## Supplementary Note 7: Limitations on $U_2(T)$

Suppose we wish to decompose $U_2(X_{\text{tot}}) = U_2(X_{\text{in}})U_2(X_{\text{enc}})$ as the sum of two parts $X_{\text{tot}} = X_{\text{in}} + X_{\text{enc}}$, as in the case where we split the operator $U_2(-3QT)$ between the client and server in order to implement $U_3(T)$. Furthermore, assume that we do not want the knowledge of $X_{\text{enc}}$ to reveal the value of $X_{\text{tot}}$. To attempt to hide the value of $X_{\text{tot}}$, we choose $X_{\text{in}}$ to be a random variable of zero mean and variance $V_{\text{in}}$. One method of characterizing the amount of information that a random variable carries about a parameter upon which its probability distribution depends on is with the Fisher information, given by

$$\mathcal{I}_X(\theta) = \mathbb{E}\left[\left(\frac{\partial}{\partial\theta}\log P(X;\theta)\right)^2\middle|\theta\right]. \tag{35}$$

Explicitly, in the case where $X_{\text{tot}} = -3QT$, the Fisher information for $X_{\text{enc}}$ has the property that $\mathcal{I}(Q) \propto T^2/V_{\text{in}}$. Notice that as $V_{\text{in}} \to 0$ the Fisher information increases without bound indicating that $X_{\text{enc}}$ allows one to make a very good estimate of the encryption parameter $Q$. However, when $V_{\text{in}} \to \infty$ then Fisher information approaches zero and the server learns no information of the encryption parameter.

## Supplementary Note 8: Equivalence of the Squeezing Gate

To demonstrate more than simply shifts in phase space, we implemented a squeezing operation, $S(r) = \exp(r(\hat{a}^2 - \hat{a}^{\dagger 2})/2)$, in the experiment. To see why this squeezing operation is as challenging as the $U_2(T)$ gate which is required for universal computation, simply notice that it is equivalent up to rotations $U_2(T) = R(\theta)S(r)R(\phi)$ [2] where $R(\theta) = \exp(i\theta\hat{a}^\dagger\hat{a})$ is generated by the free Hamiltonian. To compose gates, these additional phase shifts must be performed by the server, however to demonstrate a single $U_2(T)$ gate one can notice the following

$$C^\dagger(\alpha, T)U_2(T)D(\alpha)|\psi\rangle = C^\dagger(\alpha, T)R(\theta)S(r)R(\phi)D(\alpha)|\psi\rangle$$

$$
\begin{aligned}
&= \tilde{C}^\dagger(\alpha, T, \theta) S(r) R(\phi) \exp(\alpha \hat{a}^\dagger - \alpha^* \hat{a}) R^\dagger(\phi) R(\phi) |\psi\rangle \\
&= \tilde{C}^\dagger(\alpha, T, \theta) S(r) \exp(\alpha e^{i\phi} \hat{a}^\dagger - \alpha^* e^{-i\phi} \hat{a}) R(\phi) |\psi\rangle \\
&= \tilde{C}^\dagger(\alpha, T, \theta) S(r) D(\tilde{\alpha}) \left[ R(\phi) |\psi\rangle \right]. \qquad (36)
\end{aligned}
$$

We have defined a modified decryption operator $\tilde{C}^\dagger(\alpha, T, \theta) = C^\dagger(\alpha, T) R(\theta)$, reinterpreted the phase reference for the input state $|\psi\rangle \to R(\phi)|\psi\rangle$, and the encryption parameter has simply changed via $\tilde{\alpha} = \alpha e^{i\phi}$. A similar trick can be used to shift the rotation $R(\theta)$ through the decryption operator, which is only some displacement, to show that $\tilde{C}^\dagger(\alpha, T, \theta) = R(\theta) \mathcal{C}^\dagger(\alpha, T, \theta)$ for some displacement $\mathcal{C}$. Thus up to an appropriate redefinition of the initial and final phase references, implementation of a squeezing operation $S(r)$ is sufficient to demonstrate a $U_2(T)$ gate.

## SUPPLEMENTARY METHODS

### Determination of information content

A shot-noise limited laser at 1064 nm generated a highly coherent beam, which was split into two. One part was designated as a local oscillator (LO), the other as the signal beam. A sketch of the setup is shown in Supplementary Fig. 5.

The local oscillator was sent directly to the detection stage, while the signal beam was passed through a set of optical modulators, to be modulated in the phase and amplitude quadratures. The electronic input to the modulators in either quadrature was an addition of two signals, one representing the alphabet of the different input states of the client, the other the client's encryption noise. The noise was determined to be white within the measurement bandwidth of 1 MHz. The alphabet was recorded by the data acquisition to establish the size of the correlations. The homodyne detector output was demodulated at 10.5 MHz, with a 1 MHz lowpass filter to set the measurement bandwidth. The sampling rate was 5 MHz, with 14 bit resolution. The variance of the Gaussian distributed input state alphabet was $V_{\text{in}} = 0.6$ SNU. From the measured data we determined the variances and covariances which give an estimate for the mutual information by the formula,

$$
I(\text{server}_{\text{enc}} : \text{client}_{\text{in}}) = \frac{1}{2} \log_2 \left( \frac{V_{\text{in}}}{V_{\text{in}} - \frac{C^2}{V}} \right), \qquad (37)
$$

where $C$ is the covariance between the recorded alphabet and the signal, and $V = V_{\text{in}} + V_{\text{enc}}$, i.e. the variance of the signal and the encryption.

### Displacement gates

A shot-noise limited laser at 1064 nm generated a highly coherent beam, which was split into two. One part was designated as a local oscillator (LO), the other as the signal beam. A sketch of the setup is shown in Supplementary Fig. 6.

The local oscillator was sent directly to the detection stage, while the signal beam was passed through a set of optical modulators, to be modulated in the phase and amplitude quadratures. The electronic input to the modulators in either quadrature was an addition of two noise signals, one representing the alphabet of the different input states of the client, the other the client's encryption noise. The noise signals were determined to be white within the measurement bandwidth of 1 MHz. The state then passed through a half-wave plate and polarizing beam splitter combination which simulated an attenuation $t$ from the client to the server.

Then the state was further displaced by the server, with another signal representing the alphabet of a simple linear gate. Like the previous modulations the displacement signal was also white within the measurement bandwidth. The state was then attenuated with another polarizing beam splitter and a half-wave plate, with a setting identical to the first. This was done to simulate a state experiencing the same loss on the return trip from the server to the client. Following this final loss simulation, the state was modulated again by the client, to eliminate the encryption noise. This elimination was possible down to less than 0.2 SNU in both quadratures. This was in part made possible due to a custom-made noise generator with extremely well correlated outputs at the relevant frequencies. To obtain the right phase between the noise modulation and the cancelling modulation, a controllable phase delay between the modulations was also introduced, by using a DB64 Coax Delay Box from Stanford Research Systems from the noise generator to the modulator. The state was then interfered with the local oscillator at a 50/50 beam splitter, with the output modes subsequently detected by PIN photo diodes with a quantum efficiency of 90 %. The visibility of the interference was 92 %, due to the distortion of the spatial beam profile by the many modulators. Obtaining

this visibility was made easier by introducing a cavity to which both the signal and the local oscillator were matched, to ensure efficient overlap despite the spatial distortions.

Two types of measurements were made. The first type was where the relative phase of the local oscillator with the signal was continuously scanned by a slow function generator at 5 Hz. The outputs of the two photo detectors were subtracted and mixed down to DC using a strong electronic local oscillator at 10.5 MHz. The output of the mixer was amplified and low-pass filtered at 1 MHz before being sent to a data acquisition card (DAC) where the voltages were sampled with a rate of 5 MHz. Digitally a 10 kHz high-pass filter was implemented to dampen a strong 50 Hz modulation originating from the AC line, which was detrimental to the quality of the state reconstruction. The measurements were used to reconstruct the density matrices and Wigner functions of the input and output states using a maximum likelihood algorithm [3] and the Python module QuTiP [4].

The first measurements were used to estimate the fidelity of the decryption operation relative to an unencrypted state going through the gate and the channel, applying the definition of fidelity [5],

$$F = \mathrm{Tr}\left(\sqrt{\sqrt{\rho_0}\rho_1\sqrt{\rho_0}}\right)^2, \tag{38}$$

to the reconstructed density matrices. The different Wigner functions are shown in Supplementary Fig. 7.

The second type of measurement had the relative phase locked such that the phase quadrature was measured. Here the subtracted photo detector signals were sent to a spectrum analyzer (SA), which monitored the signal strength using a zero span trace at 10.5 MHz. This was done to easily monitor the variance of the applied Gaussian noise modulations. Though this variance was only monitored in the phase quadrature, the state reconstruction was done on-line to ensure generation of states with approximately symmetric variances. The estimation of the residual noise can be seen in Figure 7c.

### Squeezing gate

A shot-noise limited laser at 1064 nm generated a highly coherent beam, which was split into three. One part was designated as a local oscillator (LO), the second as the signal beam, and the third as the decryption beam. A sketch of the setup is shown in Supplementary Fig. 8.

The signal beam was modulated with a single displacement in $Q$ and $P$, and in addition a noise modulation generated by a noise generator. Another output of each of the noise generators, highly correlated with the first, was connected to the corresponding modulators in the decryption path. The signal beam then encountered a half-wave plate and polarizing beam splitter combination to simulate the channel, and was then forwarded through a Faraday rotator and another half-wave plate to ensure that the light was mostly in the s-polarization before entering the squeezing cavity. The light entered the resonant linear semi-monolithic cavity containing the $1 \times 2 \times 10\,\mathrm{mm}^3$ periodically poled potassium titanyl phosphate crystal pumped with a 532 nm pump beam of 7 mW through the coupling mirror. The outer crystal face was coated to have a high reflectivity of 99.95 % for both wavelengths, while a curved mirror with a radius of curvature of 20 mm, a reflectivity of 90 % for the fundamental wavelength at 1064 nm and a reflectivity of 20 % for the pump wavelength served as a piezo-tunable coupling mirror. The crystal was kept at a phase matching temperature of 36.2 °C , and most of the signal returned from where it entered, towards the Faraday rotator, but having been squeezed by performing round trips in the cavity. Before hitting the Faraday rotator, a beam sampler redirected 2 % of the light for generating an error signal for cavity and pump phase locking. Because the squeezing cavity was birefringent, the small p-polarization component was used to generate an error signal with the Hänsch-Couillaud locking technique [6]. The phase of the pump beam with respect to the signal beam was locked using a phase modulation of the signal beam at 36.7 MHz generated using the phase modulator used for encrypted input state generation.

After entering the Faraday rotator from the other direction, the beam was now reflected, rather than transmitted, on the polarizing beam splitter. It then went through a half-wave plate and polarizing beam splitter combination with the same setting as the initial one, to simulate identical channels to and from the server. It was then interfered with the decryption beam on another beam sampler, to minimize loss to the signal. The two beams were locked to destructive interference by another sideband lock, using the same 36.7 MHz sideband, with a photodiode monitoring the interference fringes in the secondary output port of the beam sampler. The offset on this error signal allowed for optimizing the relative phase between the beams, and this, in addition to the adjustable gain settings for the correlated outputs, made it possible to cancel the encryption modulation very accurately. Following the decryption operation the signal was interfered with the local oscillator for scanned homodyne detection for density matrix reconstruction. Data acquisition was the same as for the displacement gate experiment. To compute the fidelity the definition in Equation (38) was used. To reconstruct the encrypted states, which are highly thermal with a slowly decaying photon distribution, a maximum likelihood algorithm which requires a truncation of the Fock space of the density matrix is not feasible. Instead the Wigner function was obtained directly from the data with the help of the inverse Radon transform and the filtered back-projection algorithm [3]. For states of smaller magnitude where one can justify the truncation of the density matrix,

the maximum likelihood algorithm is far superior [3]. A plot of the scanned squeezing, obtained by homodyne detection, is shown in Supplementary Fig. 9.

We here list the factors contributing to optical loss in the system. Firstly, the mode matching of the input beam into the squeezer was 97 %. This was partly because of a very high sensitivity to the focus of the incoming beam, but also because, as mentioned, the Hänsch-Couillaud lock required some deviation from the ideal polarization. This intentional misalignment directly translated into loss of squeezing. Secondly, the reflected squeezed beam encountered a beam sampler, which directly produced 2 % of transmission loss, but was necessary for the locking scheme, as the pump phase error signal was not sufficiently strong in the transmission of the squeezing cavity. Further, the Hänsch-Couillaud lock clearly only works in reflection. The Faraday rotator induced 3 % loss, and the beam sampler for interfering the signal with the decryption beam produced another 2 % of loss. Lastly, the visibility of the homodyne detection was above 98 % and the photodiodes in the homodyne detector both had a quantum efficiency of 99 %.

## SUPPLEMENTARY REFERENCES


[1] S. L. Braunstein and H. J. Kimble, Phys. Rev. Lett. **80**, 869 (1998).

[2] K. Miyata, H. Ogawa, P. Marek, R. Filip, H. Yonezawa, J. I. Yoshikawa, and A. Furusawa, Physical Review A **90**, 060302(R) (2014).

[3] A. I. Lvovsky and M. G. Raymer, Reviews of Modern Physics **81**, 299 (2009).

[4] J. R. Johansson, P. D. Nation, and F. Nori, Computer Physics Communications **184**, 1234 (2013).

[5] C. Weedbrook, S. Pirandola, R. García-Patrón, N. J. Cerf, T. C. Ralph, J. H. Shapiro, and S. Lloyd, Rev. Mod. Phys. **84**, 621 (2012).

[6] T. W. Hansch and B. Couillaud, Optics Communications **35**, 441 (1980).
